# Analysis of metabolites in young and mature *Docynia delavayi* (Franch.) Schneid leaves using UPLC-ESI-MS/MS

**DOI:** 10.7717/peerj.12844

**Published:** 2022-02-04

**Authors:** Xi Xia, Can Chen, Lin Yang, Yuchang Wang, Anan Duan, Dawei Wang

**Affiliations:** 1 Key Laboratory for Forest Resource Conservation and Utilization in the Southwest Mountains of China, Ministry of Education, Southwest Forestry University, Kunming, China; 2 Key Laboratory for Forest Genetic and Tree Improvement & Propagation in Universities of Yunnan Province, Southwest Forestry University, Kunming, China

**Keywords:** *Docynia delavayi*, UPLC-ESI-MS/MS, Metabolites

## Abstract

*Docynia delavayi* (Franch.) Schneid is a plant used both as food and traditional folk medicine. The leaves of* D. delavayi* are rich in polyphenols, plants with phenolic content are known to be extremely beneficial in terms of human nutrition. In the present study, we used metabolome technology (UPLC-ESI-MS/MS) to examine the young and mature *D. delavayi* leaves on metabolites changes, which were then analyzed and compared. As a result, 477 metabolites (including 111 flavonoids, 47 others (consisted of nine vitamin, 18 saccharides and alcohols, and 20 unassigned metabolites), 71 phenolic acids, 52 amino acids and derivatives, 18 alkaloids, 61 lipids, 24 terpenoids, 33 nucleotides and derivatives, 18 lignans and coumarins, 12 tannins, 30 organic acids) were identified, of which 281 differentially accumulated metabolites, including 146 up-regulated metabolites and 135 down-regulated metabolites. The result of clustering and PCA analyses showed that young and mature leaves were separated, which indicated that there was a great difference in metabolites between young and mature leaves. Meanwhile, we also found that both young and mature leaves displayed unique metabolites with important biological functions. KEGG enrichment analysis showed that 90 of the differential metabolites were mainly concentrated in 68 KEGG pathways. The result will greatly complement the existing knowledge on the *D. delavayi* leaves for lays a foundation for subsequent development and utilization.

## Introduction

*Docynia delavayi* (Franch.) Schneid is an evergreen plant belonging to the Rosaceae family, that is widely distributed throughout southwest China (Wu, 1987). Its fruits, leaves and barks contain extensive benefits in terms of nutrients and are popular for its antioxidant activity and extremely high-quality in medicinal uses (Bao, 2001). Currently, *D. delavayi* has been made into a variety of foods, such as dried fruits, fruit wine, juice, and vinegar (Han & Ren, 2014). In the local ethnic groups of southwestern China, *Docynia* leaves are widely used as a medicine for fever, cancer, empyrosis, and rheumatism (Deng et al., 2014). It has been reported (Liu et al., 2014b) that the leaves of *D. delavayi* are rich in polyphenols and the content of polyphenol is 2-feature that of apple leaves. Moreover, *D. delavayi* has considerable leaf biomass, thus, the *D. delavayi* leaves have great potential for utilization. So far, the study of *D. delavayi* mainly involves the conventional breeding, extraction of active ingredients and functional analysis of secondary metabolites (Li, Li & Li, 2010; Liu et al., 2014a; Tai, 2006; Zhao et al., 2012).

Metabolomics is the quantitative and qualitative study of low molecular weight metabolites (Xu et al. 2020). Metabolites are the basic of biological models and enable us to gain a visual and effective understanding of biological processes and mechanisms (Chu et al., 2020). Metabonomics strategies can reveal the relationship between metabolic profiles and recessive phenotypic traits (Han et al., 2015). Of the three sequencing technologies in metabolomics, widely targeted metabolomics integrates the advantages of both targeted and non-targeted metabolomics (Sun, 2019). For the past few years, UPLC-ESI-MS (Chen et al., 2013) has been widely used as a popular technique for the analysis and identification of plant metabolites because it saves time, uses less solvent, and provides an accurate compound determination (Li et al., 2019; Wang et al., 2019a; Yang et al., 2020; Zhu, Yin & Yang, 2020; Zou et al., 2020).

At present, the research on the *D. delavayi* is still in its initial stage, especially the leaves, which have not been studied in depth. At the same time, the leaves of *D. delavayi* have a high medicinal value, so it is important to comprehensive investigation of the metabolites in *D. delavayi* leaves. To better understand the metabolic differences between the young and mature leaves of *D. delavayi*, we performed UPLC-ESI-MS/MS analysis to identify and quantify the metabolites of young and mature leaves of *D. delavayi*. The results of this study reveal the metabolic changes in the young and mature leaves of *D. delavayi* and afford vital a theory basis for the utilization of *D. delavayi* leaves.

## Material and methods

### Plant materials

*D. delavayi* leaves were collected from three uniform growth on trees and combined to make one biological replicate in Nuozhadu, Puer City, Yunnan Province, China (100°13′E, 22°34′N). The selected trees were free of pests and diseases. The plant species were identified by *Assoc. Prof.* Jianghua Liu (College of Forestry, Southwest Forestry University), and collected from the Collaborative Innovation Center of Forest Resources Breeding and Utilization in Yunnan, Southwest Forestry University, with the voucher specimen DY202035. Young leaves (first and second healthy leaves at the top of the annual branches) and mature leaves (first and second last healthy leaves at the base of annual branches) were removed from each of the three trees and the collected leaves were immediately placed in tubes and frozen in liquid nitrogen for use in subsequent experiments.

### Sample preparation and extraction

The freeze-dried *D. delavayi* leaves were crushed to powder form using a mixer containing zirconia beads for 1.5 min at 30 Hz. 100 mg weighed 100 mg of powder and added to 1.2 ml of 70% aqueous methanol for overnight extraction at 4 °C (vortex mixing 6 times during this period to improve the extraction rate). The mixed was then centrifuged at 12,000 rpm for 10 min, filtered and analysed by UPLC-MS/MS.

### UPLC conditions and ESI-QTRAP-MS/MS

The analysis of the sample extracts was performed using a UPLC-ESI-MS/MS system. It was performed by Metware Biotechnology Co., Ltd. (Wuhan, China) according to their standard procedures, as previously fully described by Gao et al. (2020). The difference was the UPLC gradient program, the flow rate and the injection volume. The gradient program was: 95:5 V/V at 0 min, 5:95 V/V at 10.0 min, 5:95 V/V at 11 min, 95:5 V/V at 11.1 min, 95:5 V/V at 14 min. The flow rate was 0.35 ml/min and the injection volume was 5 µl.

### Qualitative and quantitative determination of metabolites

Qualitative analysis of primary and mass-spectrometry data based on self-built database MWDB (Metware database) and public metabolite information database. Meanwhile, existing mass spectrometry databases (such as MassBank (http://www.massbank.jp), KNAPSAcK (http://kanaya.naist.jp/KNApSAcK) and METLIN (http://metlin.scripps.edu/index.php)) for Structural analysis of metabolites. Quantitative analysis of metabolites by triple quadrupole mass spectrometry in MRM mode as described by Yang et al. (2019). The obtained mass spectrum data were processed using Analyst software (V.1.6.2).

### Data analysis

In this study, the data were examined and their peak areas were normalized using R software. Next, the normalized data were submitted to a heatmap and the hierarchical analysis, PCA, and OPLS-DA analysis were performed by the R software. In this study, we used two screening criteria, FC (fold change) value of ≥ 2 or ≤ 0.5 and VIP value ≥ 1, to screen for differential metabolites. The significantly differential metabolites were subsequently submitted to KEGG analysis.

## Results

### Metabolic profiling

The total ion flow current (TIC) of the mass spectra obtained from the mass control samples in positive and negative ion detection modes respectively, as detected by the UPLC-MS/MS technique, were shown in Fig. S1. The high overlap of the spectra indicates that the detection method has good signal stability and reliable data results.

The metabolites of the young and mature leaves of *D. delavayi* were studied on the basis of UPLC-ESI-MS/MS and databases. Results showed that 477 metabolites were identified. The various metabolites were classified into different categories, including 111 flavonoids, 71 phenolic acids, 61 lipids, 52 amino acids and derivatives, 47 others (consisted of 9 vitamin, 18 saccharides and alcohols, and 20 unassigned metabolites), 33 nucleotides and derivatives, 30 organic acids, 24 terpenoids, 18 lignans and coumarins, 18 alkaloids and 12 tannins, as detailed in Table 1, Table S1. The analysis of the composition showed the highest content of flavonoids, accounting for 23.27% of the total metabolites.

**Table 1:** Classification of compounds in young and mature leaves of *D.delavayi*.

NO.	Metabolite class	Quantity	percentage
1	Flavonoids	111	23.27%
2	Phenolic acids	71	14.88%
3	Lipids	61	12.79%
4	Amino acids and derivatives	52	10.90%
5	Others	47	9.85%
6	Nucleotides and derivatives	33	6.92%
7	Organic acids	30	6.29%
8	Terpenoids	24	5.03%
9	Lignans and Coumarins	18	3.77%
10	Alkaloids	18	3.77%
11	Tannins	12	2.52%
	total	477	100.00%

**Figure 1:**
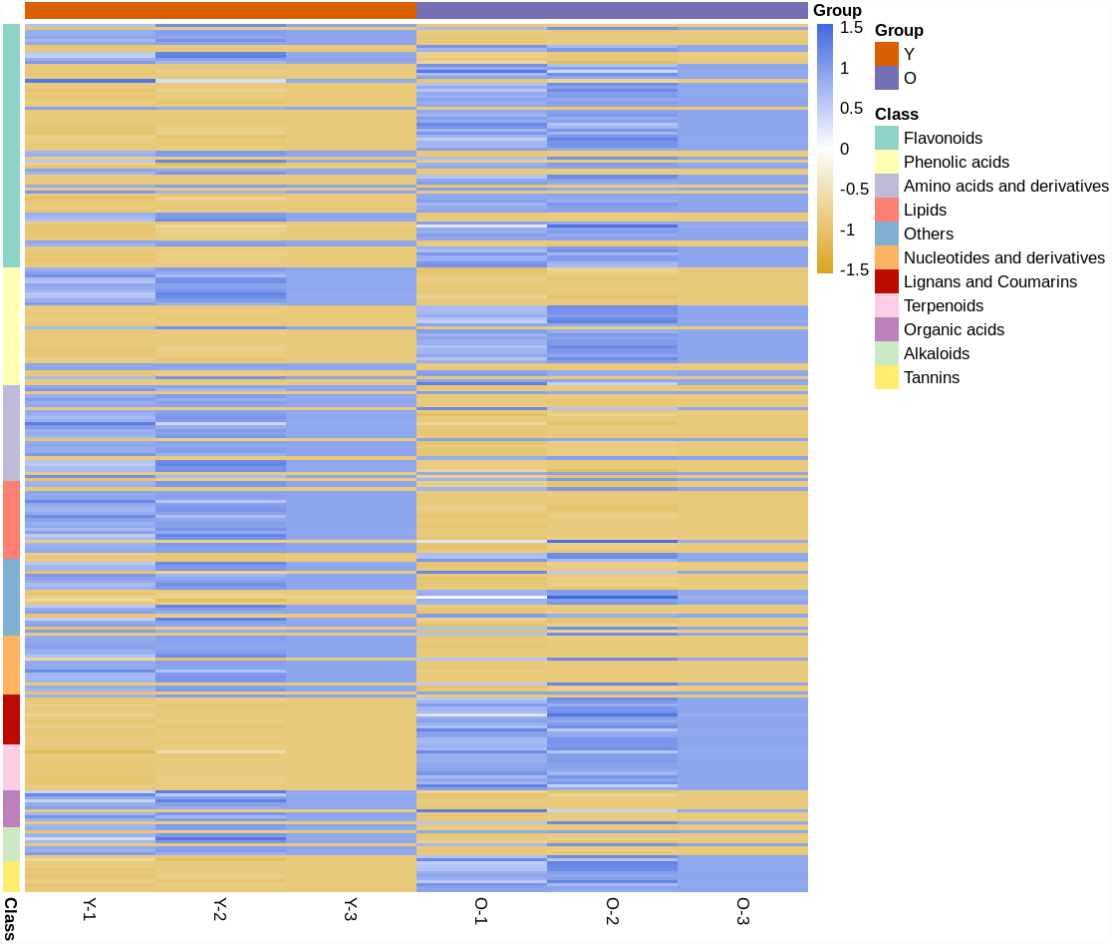
Clustering heat map of all metabolites. The horizontal axis represents the sample name and the vertical axis represents all the metabolites. The color shade reveals the content level; blue indicates that the metabolites content in the samples was higher; yellow shows that the metabolites content was lower.

In the clustering heat map (Fig. 1), it was shown by the expression levels of all metabolites that most of them were different in young and mature leaves. Compared with young leaves, most of the flavonoids and phenolic acids in the mature leaves were up-regulated. The terpenoids, tannins, and lignans and coumarins contents in mature leaves were higher than those in mature leaves. However, most of the alkaloids, others, amino acids and derivatives, organic acids and nucleotides and derivatives were down-regulated in expression in young leaves. This finding showed that *D. delavayi* mature leaves were clearly distinguished from the young leaves.

### The PCA and OPLS-DA of young and mature leaves

**Figure 2:**
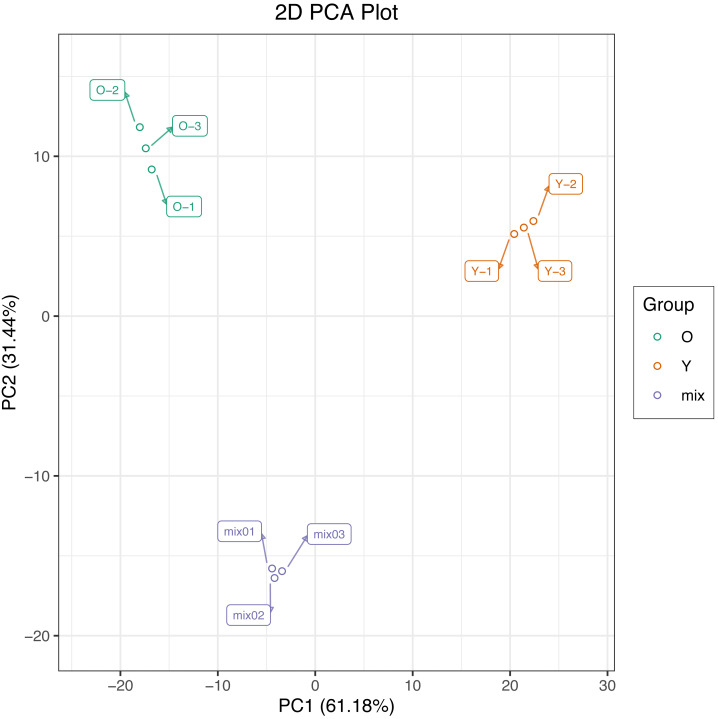
Differential metabolite analysis on the basis of principal component analysis (PCA).

In this study, we used PCA to perform multivariate statistical analysis of metabolites from six samples and extracted two principal components, PC1 and PC2, respectively, which had the cumulative contribution rates reached 92.62%. The PCA score plot show a clear metabolic difference between young and mature leaves, and the biological replicates were closely together (Fig. 2), suggesting that the experiment is reproducible and reliable.

**Figure 3:**
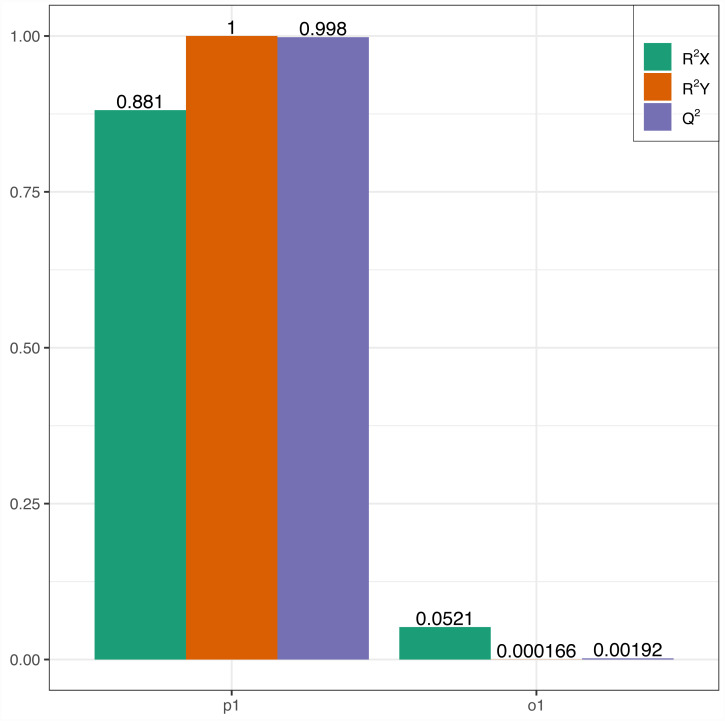
OPLS-DA permutation. R2X, R2Y represents the model interpretation rate; Q2 indicates the model predictive ability; R2Y and Q2 closer to 1 indicates that the model more stable and reliable.

In this research, samples were compared using the OPLS-DA model to evaluate the differences between Y (young leaves) and O (mature leaves). The results were R2X = 0.881, R2Y = 1, Q2 = 0.998 (Fig. 3). The Q2 values for Y and O were 0.998, which is greater than 0.9 indicating that the model was excellent and could be used for further analysis.

### Differential metabolites screening

**Figure 4:**
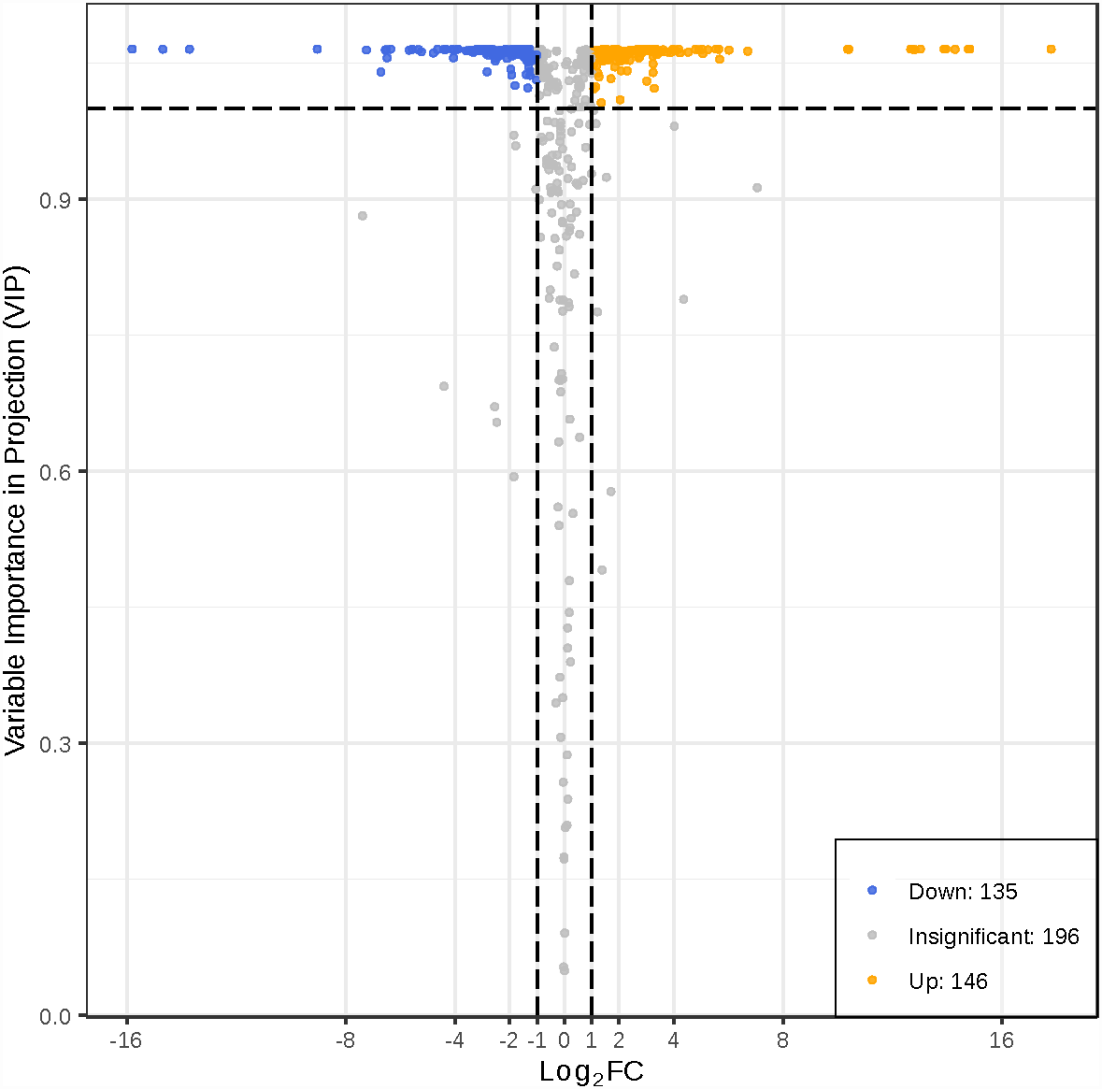
Volcano map of differential metabolites. Each point in the volcanic plot represents a metabolite, the abscissa represents the logarithm of the quantitative difference multiples of a metabolite in two samples, and the ordinate represents the variable importance in project (VIP) value. The larger the abscissa absolute value is, the more significant the differential expression is, and the more reliable the screened differential expression metabolites are. The blue dots in the figure represent down-regulated differentially expressed metabolites, the yellow dots represent up-regulated differentially expressed metabolites, and the gray dots represent metabolites detected but that are not significantly different.

To better investigate the metabolic differences between young and mature leaves of *D. delavayi* by metabolomic analysis. We screened for differential metabolites based on VIP ≥1 and FC ≥ 2 or ≤ 0.5. The significant differential metabolites among young and mature leaves are listed in Table S2. The results of differential metabolite screening for young and mature leaves were illustrated using a volcano plot (Fig. 4). Concisely, there were 281 significantly different metabolites (135 down-regulated, 146 up-regulated), the rest were no-change metabolites. These significantly different metabolites between young and mature leaves were classified into 11 different categories, including 79 flavonoid (55 up-regulated, 24 down-regulated), 38 phenolic acids (16 down-regulated,22 up-regulated), 31 amino acids and derivatives(25 down-regulated, 6 up-regulated), 19 nucleotides and derivatives(16 down-regulated, 3 up-regulated), 16 lignans and coumarins(1 down-regulated, 15 up-regulated), 25 others (15 down-regulated,10 up-regulated), 11 alkaloids (7down-regulated,4 up-regulated),12 organic acids(10 down-regulated,2 up-regulated), 25 lipids (21 down-regulated,4 up-regulated), 10 tannins and 15 terpenoids (all up-regulated).

Based on differential metabolite analysis of young and mature leaves, a total of 12 differential metabolites could only be detected in mature leaves. The 12 mature leaves specific-metabolites were myricetin 3-o-galactoside, catechin-(7,8-bc)-4 *β*-(3,4-dihydroxyphenyl)-dihydro-2-(3H)-pyranone, myricetin-3-o-(6””-malony)glucoside”, feruloylmalic acid, hesperetin O-malonylhexoside, kaempferol-3-o-rhamnoside (afzelin)(kaempferin), glutathione reduced form, feruloylsinapoyltartaric acid, aminopurine, epicatechin-epiafzelechin, apigenin-c-rhamnoside, 3 *β*,19 *α*-dihydroxyolean-12-en-28-oic acid. Syringin, n-acetyl-DL-tryptophan, quillaic acid, 4-methyl-5-thiazoleethanol, ligustilide only exist in young leaves.

### KEGG annotation and enrichment analysis

**Figure 5:**
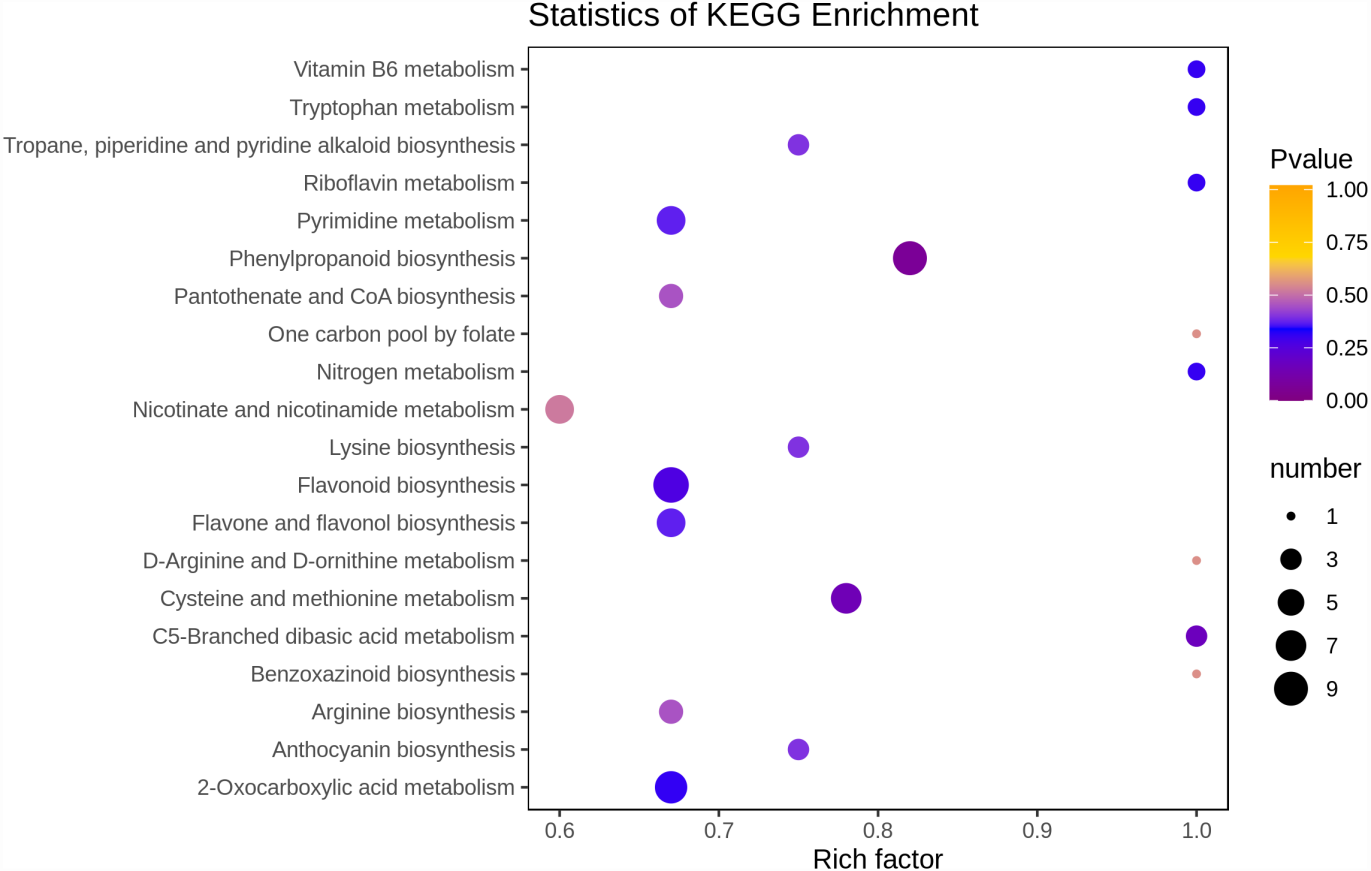
Enrichment analysis of KEGG pathway. The horizontal axis represents the enrichment factor and the vertical axis represents the pathway name. The dot color represents the *p*-value, and the dot size represents the number of differential metabolites.

In the present study, we mapped the 90 differential metabolites to the KEGG database. First, we focus on the information about metabolic pathways, and we found that most metabolites are mapped to “metabolites”. A few metabolites belong to other system information categories, such as “Genetic Information Processing” and “Environmental Information Processing”. Results of the above annotation are enriched according to the pathway types in KEGG, and the enrichment results are shown in the bubble plot in Fig. 5. A total of 68 pathways were involved in the analysis of differential metabolites between young and mature leaves, among which the top four pathways in terms of the *p*-value for metabolic pathway enrichment analysis were “Flavonoid biosynthesis”, “Cysteine and methionine metabolism”, “Phenylpropanoid biosynthesis” and “C5-Branched dibasic acid metabolism”. Among these metabolic pathways, phenylpropanoid biosynthesis and flavonoid biosynthesis contained more differential metabolites than other metabolic pathways (9 and 10, respectively).

## Discussion

In recent years, a widely targeted metabolomics method based on UPLC-ESI-MS/MS and multiple reaction monitoring (MRM) has been established as the technology has evolved. Compared to targeted metabolite detection methods, UPLC-ESI-MS/MS is characterized by high resolution, accurate characterization, short analysis times and high peak separation capacity (Farag & Shakour, 2019). For these advantages, UPLC-ESI-MS/MS is widely used for the metabolomic analyses in various plant species (Jeszka-Skowron, Zgoła-Grześkowiak & Frankowski, 2018; Matsuda et al., 2012). At present, the utilization of *D. delavayi* is mainly limited to its fruits, which are made into various food products, and the research on leaves is less, limited to the extraction of antioxidant components and determination of polyphenol content (Liu et al., 2014a; Liu et al., 2014b). Thus, this study aimed to identify the different metabolites in young and mature *D. delavayi* leaves, and the results could provide the theoretical basis for the subsequent production and utilization of *D. delavayi* leaves.

A total of 281 differential metabolites were screened by FC and VIP values. These differential metabolites best represent the differences between young and mature *D. delavayi* leaves and could provide a reference for future studies. Analysis of the differentially accumulated metabolites between the young and mature leaves showed that flavonoids are the main metabolites, of which 55 compounds were upregulated in mature leaves. It is being increasingly believed that flavonoids in leaves can promote the physiological survival of plants and protect them from fungal pathogens and UV-B radiation (Harborne & Baxter, 1999; Harborne & Williams, 2000). A previous study reported that flavonoids may function as antioxidants in response to excessive light exposure (Tattini et al., 2004). During *D. delavayi* growth, mature leaves have a longer developmental cycle than young leaves and receive external environmental stresses such as light for a longer period of time, which may be one of the reasons why most flavonoids accumulate higher in mature leaves than in young. The mature leaves of *D. delavayi* were more leathery and thicker than young, which may be related to the polyphenolic compound gallic acid in the plant (Getachew et al., 2009; Zhang et al., 2011). Studies have shown that gallic acid greatly contributes to leaf thickness and leatheriness (Kohorn & Kohorn, 2012; Rose & Lee, 2010; Wang et al., 2019b; Zhang et al., 2011).

In the metabolites of young and mature *D. delavayi* leaves, the content of cinchonain Ic, syringic sldehyde-glucoside, and L-ascorbic acid were 103, 64, and 50 times higher in mature leaves than in young leaves. We identified cinchonain Ic, syringic sldehyde-glucoside as polyphenols, which are the main antioxidant and antitumor active substances in vegetables and fruits (Gharras, 2009; Manach et al., 2004), and L-ascorbic acid as an antioxidant can synergistic effect with polyphenols to improve antioxidant efficacy (Gharras, 2009). We also detected four flavonoids (quercetin, chrysin, avicularin, and naringenin) present in *D. delavayi* leaves, which is consistent with previous studies on *D. delavayi* rhizomes (Deng et al., 2014), and all of these compounds have some antitumor activity. It could be useful for future functional and nutritional assessments of *D. delavayi* leaves. In our study, both young and mature leaves having their own unique metabolites. Differential metabolite analysis showed that most of the 12 metabolites present only in mature leaves have anti-tumor, anti-cancer, antioxidant, anti-inflammatory, anti-osteoporotic, anti-allergy, and other effects, which have high development value (Bernatova, 2018; Chen et al., 2020; García-Lafuente et al., 2009; He et al., 2020; Ke et al., 2019; Kim et al., 2004; Li et al., 2017; Wei et al., 2019; Li et al., 2019).

We also found an interesting phenomenon that terpenoids and tannins were significantly upregulated in mature leaves. Terpenoids are usually produced in vegetative tissues, flowers, and, occasionally, roots (Dudareva, Pichersky & Gershenzon, 2004). In *D. delavayi* leaves, terpenoids are mostly triterpenoids, which have the functions of resist inflammation, and inhibit the proliferation of tumor cells (Banno et al., 2005; Rufino-Palomares et al., 2013). This is similar to the previous function of specific metabolites in mature leaves, which further confirms that the content of anti-inflammatory and anti-tumour compounds is higher in mature leaves than in young. Tannins are an important bioactive class of compounds with potential antioxidant and antibacterial activities, as well as good anti-inflammatory and wound healing potential (Ambreen & Mirza, 2020; Dos Santos et al., 2017; Ogawa & Yazaki, 2018). Tannins increased as the leaves grow, all tannin compounds are upregulated in mature leaves. Reports by Hyder et al. (2002) reveal that compared to mature leaves, immature leaves of plants contained high levels of tannins, especially in stressed conditions. This is not consistent with our results and may be due to a defense mechanism that plants acquire when trying to protect themselves from excessive leaf shedding (Toit, Sithole & Vorster, 2020).

Further study of the KEGG enrichment analysis showed that the most significant of phenylpropanoid biosynthesis was found in the comparison groups between young and mature leaves. A total of nine metabolites were enriched to this pathway and their structures metabolites are shown in Fig. S2, with most of the differential metabolites (l-phenylalanine, caffeic acid, ferulic acid, coniferyl alcohol, syringin, sinapaldehyde) being down-regulated in mature leaves (Fig. S3). This may be due to the increase in secondary metabolites such as phenolic acids that were critical for environmental adaptation and plant survival during early nutrition (Aidoo et al., 2016; Zhang et al., 2017).

## Conclusions

In the present study, we successfully performed UPLC-ESI-MS/MS-based metabolic analysis to compare metabolites of young and mature leaves of *D. delavayi* in a systematic method. A total of 477 metabolites were detected, 281 of which were differential metabolites. Differential metabolite analysis showed that flavonoids were the predominant metabolites, with most flavonoids, terpenoids and tannins being up-regulated in mature leaves. In addition, we identified 12 compounds present only in mature leaves, all of which have some antioxidant, anti-inflammatory and anti-cancer effects. Overall, this study contributes to the understanding the composition of *D. delavayi* leaves metabolites and also provides a reference for the future medical use and development of *D. delavayi* leaves.

##  Supplemental Information

10.7717/peerj.12844/supp-1 Supplemental Information 1 All metabolites of mature and old *D. delavayi* leaves

10.7717/peerj.12844/supp-2 Supplemental Information 2 Differential metabolites of young and mature D. delavayi leaves

10.7717/peerj.12844/supp-3 Supplemental Information 3 TIC overlap map of QC samples A: positive ion mode; B: negative ion mode

10.7717/peerj.12844/supp-4 Supplemental Information 4 Structure of metabolites enriched to the phenylpropanoid biosynthesis pathway

10.7717/peerj.12844/supp-5 Supplemental Information 5 Phenylpropanoid biosynthesis pathways in *D. delavayi* leaves. (comparison between young and mature leaves) The red dots indicate up-regulated metabolites, green dots indicate down-regulated metabolites, blue dots no significant change in metabolites.

## Additional Information and Declarations

### Data Availability

The following information was supplied regarding data availability: The raw data is available in the Supplemental File.
