# Supplementary figures and images for: Analysis of metabolites in young and mature Docynia delavayi (Franch.) Schneid leaves using UPLC-ESI-MS/MS
Source: PeerJ. 2022 Feb 4;10:e12844. doi: 10.7717/peerj.12844 (PMC8820213; doi:10.7717/peerj.12844)

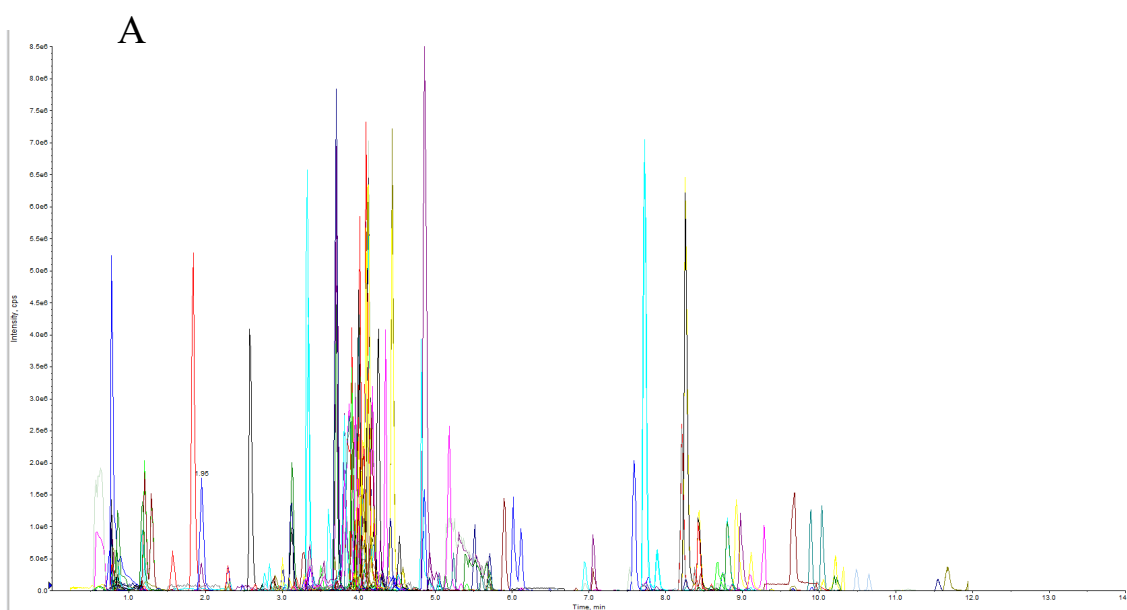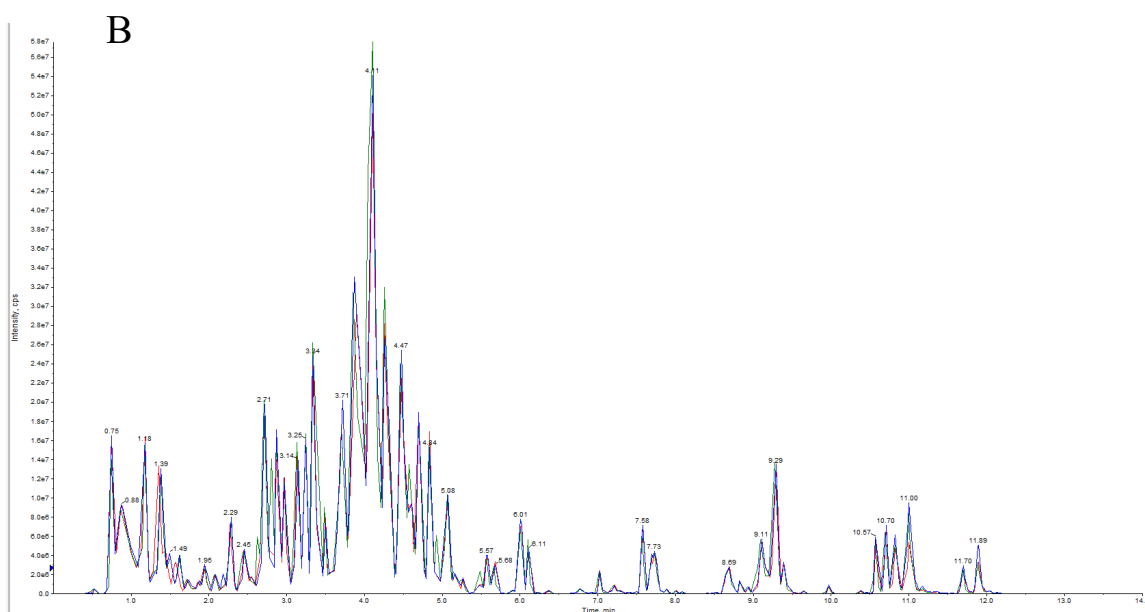

Supplement: Supplemental Information 3 — A: positive ion mode; B: negative ion mode [file peerj-10-12844-s003.pdf]
