# Supplementary material for: Analysis of metabolites in young and mature Docynia delavayi (Franch.) Schneid leaves using UPLC-ESI-MS/MS
Source: PeerJ. 2022 Feb 4;10:e12844. doi: 10.7717/peerj.12844 (PMC8820213; doi:10.7717/peerj.12844)
Supplement: Supplemental Information 4 [file peerj-10-12844-s004.pdf]

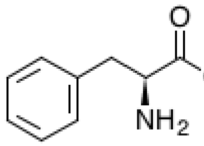

C00079

A: L-Phenylalanine

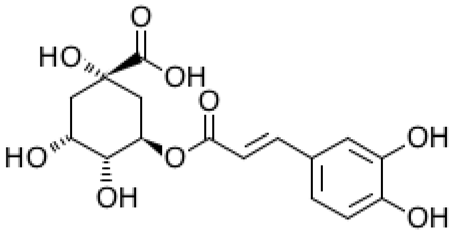

C00852

B: Chlorogenic acid

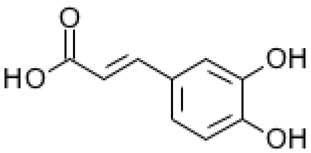

C01197

C: Caffeic acid

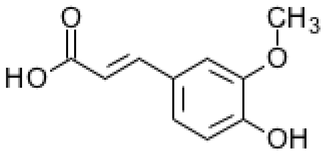

C01494

D: Ferulic acid

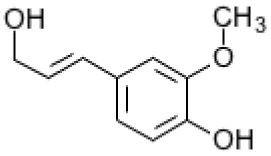

C00590

E: Coniferol

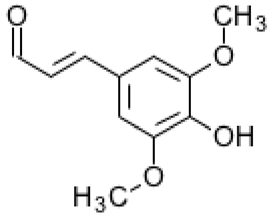

C05610

F: Sinapaldehyde

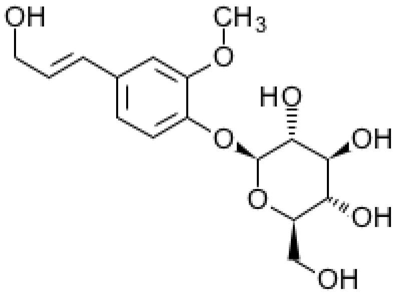

C00761

G: Coniferin

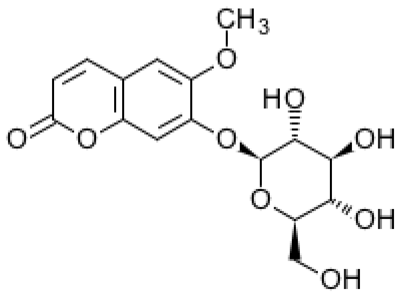

C01527

H: Scopolin

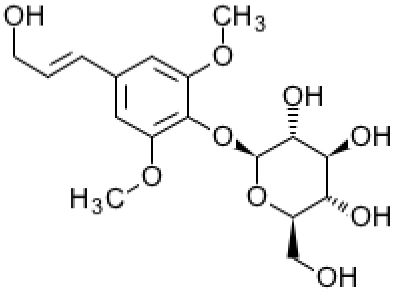

C01533

I: Syringin
