# Supplementary material for: Analysis of metabolites in young and mature Docynia delavayi (Franch.) Schneid leaves using UPLC-ESI-MS/MS
Source: PeerJ. 2022 Feb 4;10:e12844. doi: 10.7717/peerj.12844 (PMC8820213; doi:10.7717/peerj.12844)
Supplement: Supplemental Information 5 — The red dots indicate up-regulated metabolites, green dots indicate down-regulated metabolites, blue dots no significant change in metabolites. [file peerj-10-12844-s005.pdf]

PHENYLPROPANOID BIOSYNTHESIS

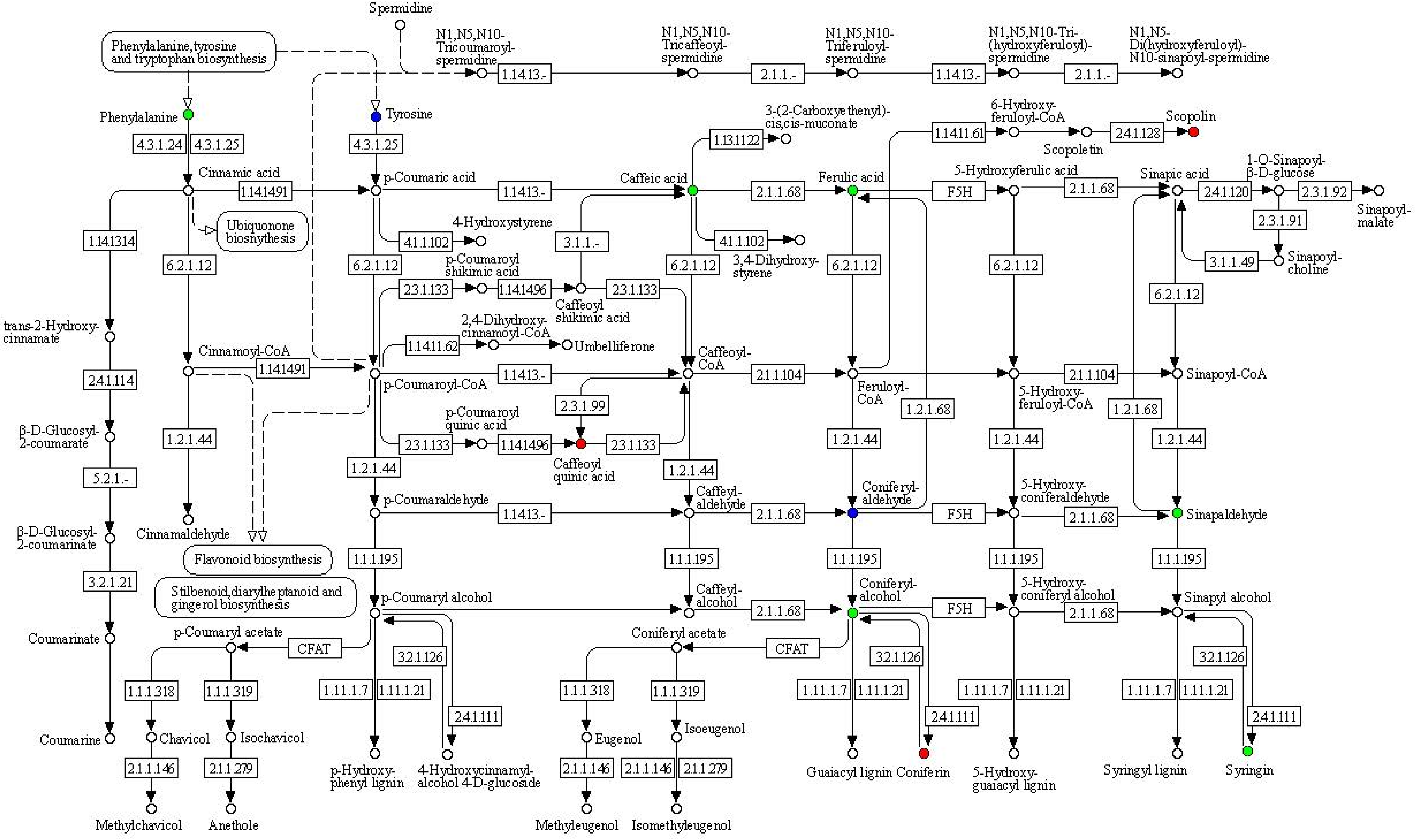

00940 3/25/20

(c) Kanehisa Laboratories
